# Match running performance characterizing the most elite soccer match-play

**DOI:** 10.5114/biolsport.2023.124847

**Published:** 2023-02-03

**Authors:** Toni Modric, Sime Versic, Ryland Morgans, Damir Sekulic

**Affiliations:** 1 Faculty of Kinesiology, University of Split, Split, Croatia; 2 Football Performance Hub, University of Central Lancashire, Preston PR1 2HE, UK; 3 High Performance Sport Center, Croatian Olympic Committee, Zagreb, Croatia

**Keywords:** Physical performance, Elite players, Football, Match analysis, Big five league

## Abstract

In order to identify match running performance (MRP) characterizing the most elite soccer match-play, this study aimed to examine position-specific differences in the MRP of players competing in “big five” (BFLTs) and “non-big five” league teams (N-BFLTs). The data were obtained from 24 teams (BFLTs; n = 14, N-BFLTs; n = 10) during the UEFA Champions League (UCL) matches (n = 20) in the 2020/21 season using a semiautomatic video system. The differences in MRP between BFLTs and N-BFLTs, while controlling for contextual factors, were examined using linear mixed model. No differences in overall MRP between fullbacks, central midfielders, wide midfielders and forwards from BFLTs and their peers from N-BFLTs were found, while only central defenders from BFLTs covered more high-intensity running than central defenders from BFLTs (moderate effects size). For players on all playing positions from BFLTs, total- and low-intensity distance covered were lower in offensive phase of game and greater in defensive phase of game compared to their peers from N-BFLTs (all large effect sizes). This study demonstrated that the most elite match-play in soccer is characterized by increased efforts in defensive phase of game, and decreased efforts in offensive phase of game. Soccer training programmes should be adapted accordingly.

## INTRODUCTION

Match performance in soccer is highly dependent on the interactions between technical, tactical and physical performance [1]. Although technical–tactical performances are considered decisive for success in soccer [2], players must still be able to handle the high physical demands that contemporary soccer requires [3]. In addition, detailed knowledge about the physical performance of professional soccer match play is required to construct optimal training programs to respond to these needs [4]. For example, the distances covered by players in a match, according to their positions, can be used to prescribe more specific training or to consider new ways to improve the efficiency of team training [5]. For these reasons, many techniques (e.g., global and local positioning systems or multi-camera optical systems) have been used to establish the physical profiles of soccer players by quantifying match running performance (MRP) [6–10].

Previous research analysing MRP in soccer demonstrated that elite players can cover between 9 and 14 km, performing 0.7–3.9 km of high-speed distance and 0.2–0.6 km of sprint distance [11]. These performances are primarily determined by the different playing positions of the players in the match [12]. Specifically, the majority of studies reported that central midfielders cover the greatest overall distance and central defenders cover the lowest overall distance of all the playing positions [13, 14]. In addition, studies have typically reported greater high-intensity running distances (> 5.5 m/s) for wide midfielders and fullbacks compared to other positions, with central defenders performing the lowest high-intensity running distance among elite players [13, 14]. In addition to playing position, the MRP of soccer players can be further determined using specific match-related factors, such as match location or match outcome [15]. Controlling the influence of these factors when examining the MRP of soccer players is essential.

Soccer is no doubt one of the most popular sports in the world, played in more than 200 countries, with, currently, more than 500 million active players, of whom 300 million are registered soccer club members [16]. The most prestigious competition in soccer is the Union of European Football Associations (UEFA) Champions League (UCL) [17]. Millions of soccer supporters in Europe and throughout the world are interested in the games and title winners [17]. Together with the financial perspective, this attracts top-level soccer players from all over the world to join teams competing in the UCL. As a result, teams that compete in the UCL consist of some of the most elite soccer players in the world. Analyzing the MRP of such players can help to constitute activity profiles which can be used by coaches and players for reaching the most elite match-play [18]. Therefore, it is not surprising that researchers have put forth great effort in providing knowledge about the MRP of players competing in UCL [3, 11, 19–22].

Briefly, Di Salvo et al. provided useful insight into position-specific sprinting activities during UCL matches [20]. Minano-Espin et al. analysed the high-intensity running patterns of UCL players according to the quality of the opposition [21]. The focus of Bradley et al. was on the gender differences between UCL players, indicating large differences in the physical indicators between male and female players [19]. Modric et al. evaluated position-specific MRP (overall and specifically for the offensive and defensive phases of the game) according to success in the UCL group stage [3, 22]. In addition, the same authors have provided information on match-related factors affecting MRP in UCL matches, demonstrating the influence of match outcome and match location over variables determining the intensity and volume of the match, respectively [11].

Although these studies provided some valuable information on the MRP in elite soccer, the real knowledge on MRP characterizing the most elite match-play is still limited. Specifically, despite the high standards of the teams competing in the UCL, representing the cream of each national league [20], the samples used in all the aforementioned studies also comprised teams from lower-standard national leagues [3, 19, 20,22]. Specifically, Di Salvo et al.’s sample included teams from the UEFA European League, Bradley et al.’s sample included the top 15 ranked teams in Europe, and Modric et al. analysed group-stage matches that typically include lower-standard teams [3, 19, 20, 22]. Furthermore, none of these studies controlled for the influence of contextual factors that may significantly affect the conclusions [15].

For all these reasons, further research, utilizing a more complex statistical approach that controls for contextual factors, is needed to provide true knowledge on the MRP of the most elite soccer players. According to the UEFA, Spanish La Liga, English Premier League, Italian Serie A, German Bundesliga and French Ligue 1 are the top five ranked professional soccer leagues [23]; therefore, players competing in UCL which national competitions play in one of these leagues can be considered as most elite soccer players in the world. Analysing the differences in MRP between such players and their peers from lower-standard teams may help soccer coaches to identify critical the MRP required for reaching elite match-play [18], while at the same time can be used to adapt soccer training programs accordingly. Therefore, the main objective of this study was to examine the position-specific differences in the MRP of UCL players from teams competing in “big five” or “non-big five” leagues.

## MATERIALS AND METHODS

### Participants and study design

The sample comprised 547 individual match observations of 378 outfield players who were members of 24 teams that competed in the group stage of the UCL in the 2020/21 season. Depending on whether they played national competitions in “big five” or “non-big five” leagues, teams were classified into two groups as: big five league teams (BFLTs; n = 14) and non-big five league teams (N-BFLTs; n = 10). The BFLTs included teams from Spanish La Liga (n = 4), Italian Serie A (n = 3), German Bundesliga (n = 3), England Premier League (n = 2) and France Ligue 1 (n = 2). N-BFLTs included teams from Russian Premier Liga (n = 3), Ukrainian First League (n = 2), Portuguese Primeira Liga (n = 1), Hungarian OTP Bank Liga (n = 1), Belgian First Division A (n = 1), Super League Greece (n = 1) and Austrian Football Bundesliga (n = 1).

All data were obtained from 20 matches from groups A (n = 3), B (n = 3), C (n = 4), E (n = 4), F (n = 3) and G (n = 3). Matches that included a player dismissal (red card) were not observed, and only results from players who participated in the whole match were analysed [20]. As a result, 244 match observations (BFLTs; n = 89, N-BFLTs; n = 155) were retrieved and classified according to soccer-specific playing positions as follows: central defender (CD; n = 79), fullback (FB; n = 65), central midfielder (CM; n = 55), wide midfielder (i.e., winger) (WM; n = 28) and forward (FW; n = 17).

Players’ and teams’ identities were anonymized per the principles of the Declaration of Helsinki to ensure confidentiality. The investigation was approved by the local university ethics board, while written permission for the data used was obtained from InStat Limited (Limerick, Republic of Ireland, 5 June 2021).

### Procedures

All MRP data were recorded using a multicamera, semiautomatic optical tracking system (InStat Fitness, Instat Limited, Limerick, Republic of Ireland) with a sampling frequency of 25 Hz. The system passed the official Fédération Internationale de Football Association (FIFA) test protocol for electronic and performance tracking systems (EPTS) (authorization number: 1007382), demonstrating high levels of absolute and relative reliability [11].

The MRPs were observed separately for (i) overall match, (ii) offensive phase of the game and (iii) defensive phase of the game. The MRP in the offensive phase of the game was quantified when the team had the ball in their possession, while the MRP in the defensive phase of the game was quantified when the opponent had the ball in their possession [24].

The MRP variables included total distance covered (TD) (m), low-intensity running (LIR) (≤ 4 m/s) (m), moderate-intensity running (MIR) (4–5.5 m/s) (m), high-intensity running (HIR) (≥ 5.5 m/s) (m), TD in the offensive phase of the game (TD-O) (m), LIR in the offensive phase of the game (LIR-O) (m), MIR in the offensive phase of the game (MIR-O) (m), HIR in the offensive phase of the game (HIR-O) (m), TD in the defensive phase of the game (TD-D) (m), LIR in the defensive phase of the game (LIR-D) (m), MIR in the defensive phase of the game (MIR-D) (m), HIR in the defensive phase of the game (HIR-D) (m) [11, 22].

### Statistics

The normality of the distributions was checked with the Kolmogorov–Smirnov test, and the descriptive statistics included the means ± standard deviations. The homoscedasticity of all variables was confirmed with Levene’s test. Linear mixed model was adjusted to examine the differences in MRPs (i.e., dependent variables) of players from BFLTs and N-BFLTs. For this purpose, a dummy variable “league” (coded as “1” if the team was a member of the “big five” leagues and “0” if the team was not a member of the “big five” leagues) was created and introduced in the model as fixed effect. Considering the very recent study which demonstrated that match outcome and match location may influence MRP in UCL [11], differences in MRP between BFLTs and N-BFLTs were examined while controlling for match location and match outcome. For this purpose, dummy variables “match outcome” (not-win/win) and “match location” (home/away) were created and introduced into the model as fixed effects. By adding these fixed effects, the pure differences between players from BFLTs and N-BFLTs can be more accurately assessed. To account for the repeated measurements, players’ and teams’ identities were modelled as random effects. The assumptions of homogeneity and normal distributions of residuals were without revealing specific problems. The main effect (i.e., BFLTs vs N-BFLTs) comparisons were summarized using least significant difference (LSD) [25]. The t-statistics from the mixed models were converted to effect sizes (Cohen’s d) [26], and interpreted as follows: trivial (< 0.2), small (> 0.2–0.5), moderate (> 0.5–0.8) and large (> 0.8) [27]. The 95% confidence intervals were computed to assess the precision of the estimates. To determine most important metrics distinguishing most elite soccer players (i.e., from BFLTs) and their counterparts from lower-standard teams (i.e., from N-BFLTs), multivariate differences in MRP between BFLTs vs N- BFLTs were analyzed by canonical discriminant analysis. Comparison for MRPs between won and lost matches was examined using one-way analysis of variance. Differences in ball possession between BFLTs and N-BFLT were examined by Mann-Whitney U Test to support introduced assumptions. All the analyses were performed using the SPSS software (IBM, SPSS, Version 25.0).

## RESULTS

The CDs from N-BFLTs covered less HIR than CDs from BFLTs (586 and 716 m, respectively; effect size (ES) (95% confidence interval [CI]) = -0.64 (-1.21; -0.07), while CMs, FBs, FWs, and WMs covered similar HIR irrespective playing in N-BFLTs or BFLTs. Players on all other playing positions covered similar TD (CDs: 10,165 m and 10,240 m, respectively; CMs: 11,815 and 11,967, respectively; FBs: 10,840 m and 11,069, respectively; FWs: 10,854 m and 10,318 m, WMs: 11,247 m and 10,843 m, respectively), LIR (CDs: 8013 m and 7948 m respectively; CMs: 8474 m and 8470 m, respectively; FBs: 8063 m and 8076 m, respectively; FWs: 8017 m and 7830 m, respectively; WMs: 8187 m and 7941 m, respectively), and MIR (CDs: 1569 m and 1577 m, respectively; CMs: 2499 m and 2504 m, respectively; FBs: 1843 m and 1925 m, respectively; FWs: 1803 m and 1679 m, respectively; WMs: 2048 m and 1780 m, respectively). As covariates, match outcome and match location did not impact TD, LIR, MIR and HIR (tables 1 and 2).

**TABLE 1:** Descriptive statistics for MRP of players from BFLTs and N-BFLTs while controlling influence of match outcome and match location (data are given as mean ± SD).

	Central defenders		Central midfielders		Fullbacks		Forwards		Wide midfielders	

	N-BFLT	BFLT	N-BFLT	BFLT	N-BFLT	BFLT	N-BFLT	BFLT	N-BFLT	BFLT
TD (m)	10165 ± 976	10240 ± 758	11815 ± 748	11967 ± 642	10840 ± 936	11069 ± 756	10854 ± 1045	10318 ± 785	11247 ± 911	10843 ± 686
LIR (m)	8013 ± 701	7948 ± 550	8474 ± 396	8470 ± 359	8063 ± 613	8076 ± 502	8017 ± 590	7830 ± 443	8187 ± 581	7941 ± 437
MIR (m)	1569 ± 400	1577 ± 313	2499 ± 487	2504 ± 427	1843 ± 407	1925 ± 326	1803 ± 481	1679 ± 361	2048 ± 423	1780 ± 346
HIR (m)	586 ± 284	716 ± 221	857 ± 328	1001 ± 277	954 ± 287	1072 ± 235	1038 ± 308	811 ± 230	1070 ± 272	1113 ± 204

TD-O (m)	4163 ± 869	3119 ± 732	5031 ± 938	3660 ± 831	4366 ± 873	3148 ± 776	3971 ± 928	2630 ± 716	4691 ± 1198	2972 ± 898
LIR-O (m)	3086 ± 711	2016 ± 637	3183 ± 664	2056 ± 618	2997 ± 709	1963 ± 631	2793 ± 654	1829 ± 504	3175 ± 828	1879 ± 648
MIR-O (m)	747 ± 227	674 ± 181	1368 ± 354	1076 ± 312	891 ± 201	718 ± 170	782 ± 280	550 ± 213	1051 ± 317	672 ± 256
HIR-O (m)	366 ± 152	420 ± 122	498 ± 143	520 ± 135	464 ± 126	469 ± 112	405 ± 145	252 ± 112	446 ± 165	420 ± 129

TD-D (m)	2965 ± 802	4097 ± 646	3382 ± 711	5018 ± 619	3218 ± 865	4572 ± 705	3518 ± 637	4635 ± 478	3289 ± 728	4700 ± 547
LIR-D (m)	2318 ± 641	3305 ± 519	2333 ± 581	3584 ± 504	2202 ± 551	3243 ± 469	2280 ± 571	3310 ± 427	2111 ± 526	3329 ± 412
MIR-D (m)	540 ± 248	607 ± 193	796 ± 278	1065 ± 237	624 ± 284	862 ± 227	717 ± 208	875 ± 157	696 ± 220	826 ± 186
HIR-D (m)	121 ± 160	185 ± 124	258 ± 248	363 ± 209	370 ± 198	471 ± 160	516 ± 209	450 ± 157	491 ± 230	542 ± 180

N-BFLTs – non-big five league teams, BFLTs – big five league teams; TD – total distance, LIR – low intensity running, MIR – moderate intensity running, HIR – high intensity running; TD-O – total distance in offensive phase of game, LIR-O – low intensity running in offensive phase of game, MIR-O – moderate intensity running in offensive phase of game, HIR-O – high intensity running in offensive phase of game; TD-D – total distance in defensive phase of game, LIR-D – low intensity running in defensive phase of game, MIR-D – moderate intensity running in defensive phase of game, HIR-D – high intensity running in defensive phase of game.

**TABLE 2:** The effect of league on overall MRP while controlling influence of match outcome and match location.

	Central defenders		Central midfielders		Fullbacks		Forwards		Wide midfielders	

	β	95%CI	β	95%CI	β	95%CI	β	95%CI	β	95%CI
TD										
Intercept	10297	(9967;10627)	12028	(11649;12408)	11289	(10876;11701)	10657	(9640;11674)	10930	(10325;11534)
League (N-BFLT)	-75	(-470;321)	-152	(-535;231)	-229	(-658;200)	536	(-462;1534)	404	(-256;1064)
Match outcome (not win)	35	(-214;284)	35	(-287;356)	-254	(-621;113)	-535	(-1631;561)	181	(-502;865)
Location (home)	-149	(-354;56)	-157	(-435;121)	-185	(-492;122)	-143	(-1084;798)	-355	(-883;172)

LIR										
Intercept	7991	(7738;8244)	8609	(8384;8834)	8278	(7995;8561)	8026	(7451;8600)	8034	(7649;8419)
League (N-BFLT)	65	(-220;351)	4	(-203;211)	-13	(-296;271)	187	(-377;751)	245	(-175;666)
Match outcome (not win)	26	(-187;238)	-170	(-373;32)	-269	(-527;-12)	-286	(-905;333)	61	(-372;494)
Location (home)	-112	(-291;68)	-109	(-293;75)	-134	(-354;85)	-105	(-637;427)	-246	(-581;89)

MIR										
Intercept	1615	(1471;1759)	2483	(2222;2745)	1985	(1812;2157)	1823	(1356;2290)	1880	(1612;2148)
League (N-BFLT)	-8	(-170;155)	-5	(-256;247)	-82	(-267;103)	124	(-334;583)	268	(-44;581)
Match outcome (not win)	-56	(-177;65)	91	(-139;322)	-85	(-234;64)	-154	(-657;350)	-85	(-479;309)
Location (home)	-20	(-122;81)	-49	(-255;156)	-34	(-157;88)	-135	(-567;298)	-115	(-382;153)

HIR										
Intercept	668	(569;768)	954	(799;1109)	1037	(905;1170)	805	(506;1104)	1089	(910;1267)
League (N-BFLT)	**-130**	**(-245;-15)**	-144	(-311;23)	-118	(-250;14)	227	(-66;520)	-43	(-240;153)
Match outcome (not win)	71	(-8;151)	77	(-45;198)	74	(-46;194)	-93	(-415;229)	86	(-125;296)
Location (home)	24	(-42;90)	18	(-83;119)	-4	(-105;98)	104	(-174;381)	-36	(-194;121)

β – estimate, CI – confidence interval; TD – total distance, LIR – low intensity running, MIR – moderate intensity running, HIR – high intensity running. Bold text denotes statistical significance of p < 0.05.

Players from N-BFLTs on all playing positions covered more TD-O (CDs: 4163 m and 3119 m, respectively; ES (95%CI) = 1.64 (0.98, 2.28), CMs: 5031 m and 3660 m, respectively; respectively; ES (95%CI) = 1.81, (1.06, 2.55), FBs: 4366 m and 3148 m, respectively; ES (95%CI) = 1.49 (0.92, 2.06), FWs: 3971 m and 2630 m, respectively; ES (95%CI) = 1.81 (0.50, 3.07), WMs: 4691 m and 2972, respectively; ES (95%CI) = 1.69 (0.74, 2.61)) and LIR O (CDs: 3086 m and 2016 m; respectively; ES (95%CI) = 1.60 (1.08, 2.12), CMs: 3183 m and 2056 m, respectively; ES (95%CI) = 2.22 (1.36, 3.07), FBs: 2997 m and 1963 m, respectively; ES (95%CI) = 1.56 (0.98, 2.13), FWs: 2793 m and 1829 m, respectively; ES (95%CI) = 1.85 (0.53, 3.12), WMs: 3175 m and 1879 m, respectively; ES (95%CI) = 1.78 (0.83, 2.71)).

In addition, CMs (1368 m and 1076 m, respectively; ES (95%CI) = 1.00 (0.35, 1.65)), FBs (891 m and 718, respectively; ES (95%CI) = 1.08 (0.46, 1.69)), and WMs (1051 m and 672 m, respectively; ES (95%CI) = 1.39 (0.47, 2.28)) from N-BFLTs covered more MIR-O than their counterpart from BFLTs. The FWs (405 m and 252 m, respectively; ES (95%CI) = 1.32 (0.11, 2.50) from N-BFLTs more HIR-O than FWs from BFLTs. As covariates, match outcome and match location did not impact TD, LIR, MIR and HIR (tables 1 and 3).

**TABLE 3:** The effect of league on MRP in offensive phase of game while controlling influence of match outcome and match location.

	Central defenders		Central midfielders		Fullbacks		Forwards		Wide midfielders	

	β	95%CI	β	95%CI	β	95%CI	β	95%CI	β	95%CI
TD-O										
Intercept	**3020**	**(2643;3396)**	**3555**	**(3041;4068)**	**3288**	**(2819;3758)**	**2981**	**(2079;3883)**	**3297**	**(2511;4082)**
League (N-BFLT)	1045	(678;1411)	1370	(884;1857)	1218	(801;1635)	1341	(453;2229)	1719	(856;2583)
Match outcome (not win)	229	(-125;583)	296	(-160;752)	-73	(-511;364)	-119	(-1104;866)	-76	(-1017;866)
Location (home)	-31	(-345;282)	-85	(-494;325)	-207	(-596;183)	-582	(-1397;233)	-575	(-1270;121)

LIR-O										
Intercept	1961	(1625;2298)	2045	(1654;2436)	2099	(1717;2480)	2032	(1396;2667)	2205	(1628;2781)
League (N-BFLT)	**1070**	**(763;1378)**	**1126**	**(771;1481)**	**1034**	**(695;1372)**	**964**	**(339;1590)**	**1296**	**(684;1908)**
Match outcome (not win)	186	(-136;509)	99	(-258;455)	-73	(-429;282)	44	(-649;738)	-148	(-805;510)
Location (home)	-77	(-367;212)	-76	(-407;254)	-199	(-515;118)	-451	(-1025;123)	-504	(-1012;4)

MIR-O										
Intercept	654	(567;741)	1018	(826;1209)	770	(670;869)	635	(363;907)	769	(567;970)
League (N-BFLT)	72	(-20;165)	**292**	**(110;475)**	**173**	**(79;267)**	232	(-35;499)	**379**	**(145;612)**
Match outcome (not win)	0	(-77;77)	139	(-30;307)	-56	(-148;35)	-44	(-338;251)	-44	(-295;206)
Location (home)	40	(-26;106)	-22	(-172;129)	-46	(-126;34)	-127	(-377;123)	-148	(-330;34)

HIR-O										
Intercept	376	(317;435)	470	(384;555)	427	(359;495)	305	(164;446)	340	(225;456)
League (N-BFLT)	-54	(-116;9)	-22	(-98;54)	-5	(-65;55)	**154**	**(15;292)**	26	(-96;148)
Match outcome (not win)	46	(-7;99)	64	(-14;142)	53	(-10;116)	-114	(-268;40)	108	(-24;239)
Location (home)	42	(-4;88)	36	(-36;108)	31	(-25;87)	8	(-119;135)	51	(-51;152)

β – estimate, CI – confidence interval; TD-O – total distance in offensive phase of game, LIR-O – low intensity running in offensive phase of game, MIR-O – moderate intensity running in offensive phase of game, HIR-O – high intensity running in offensive phase of game. Bold text denotes statistical significance of p < 0.05.

Players from N-BFLTs on all playing positions covered less TD-D (CDs: 2965 m and 4097 m, respectively; ES (95%CI) = -1.80 (-2.40, -1.19), CMs: 3382 m and 5018, respectively; ES (95%CI) = -2.84 (-3.70, -1.96), FBs: 3218 m and 4572 m, respectively; ES (95%CI) = -2.14 (-2.89, -1.37), FWs: 3518 m and 4635 m, respectively; ES (95%CI) = -2.33 (-3.76, -0.83), WMs: 3289 m and 4700 m, respectively; ES (95%CI) = 2.27 (1.23, 3.28)) and LIR-D (CDs: 2318 m and 3305 m, respectively; ES (95%CI) = -2.09 (-2.76, -1.40), CMs: 2333 m and 3584 m, respectively; ES (95%CI) = -2.69 (-3.54, -1.83), FBs: 2202 m and 3243 m, respectively; ES (95%CI) = -2.48 (-3.27, -1.68), FWs: 2280 m and 3310 m, respectively; ES (95%CI) = -2.38 (-3.83, -0.88), WMs: 2111 m and 3329 m, respectively; ES (95%CI) = -2.64 (-3.71, -1.53)).

In addition, CMs from N-BFLTs covered less MIR-D than CMs from BFLTs (796 m and 1065 m, respectively; ES (95%CI) = -1.23 (-1.92, -0.54)), while FBs from N-BFLTs covered less MIR-D (624 m and 862 m, respectively; ES (95%CI) = -1.12 (-1.75, -0.48)) and HIR-D (370 m and 471 m, respectively; ES (95%CI) = -0.69 (-1.31, -0.06)) than their counterparts from BFLTs. As covariates, match outcome had an influence on TD-D for CMs, while match location did not impact TD-D, LIR-D, MIR-D and HIR-D (table 1 and 4).

Table 5 presents a discriminant canonical analysis of multivariate differences in MRP between N-BFLTs and BFLTs. The results show significant differentiation between N-BFLTs and BFLTs in MRP for all players (CanR = 0.80). The LIR-D most greatly contributed to the differentiation for all players (correlation with the discriminant function r = 0.87), with higher occurrence in BFLTs. A discriminant function correctly classified 91% of the cases (94% and 87% for N-BFLTs and BFLTs, respectively). Analysing soccer-specific playing position, significant differentiation between N-BFLTs and BFLTs in MRP was evidenced for CDs (CanR = 0.78), CMs (CanR = 0.79), FBs (CanR = 0.89), and WMs (CanR = 0.80) (i.e., it was not possible analyse FWs due to the limited sample for this position). The LIR-D was highly correlated with discriminant functions for all of them (r = 0.78–0.89), with higher occurrence in BFLTs. The discriminant functions correctly classified 91% of the cases for CDs and CMs, 93% for FBs, and 100% for WMs.

**TABLE 4:** The effect of league on MRP in defensive phase of game while controlling influence of match outcome and match location.

	Central defenders		Central midfielders		Fullbacks		Forwards		Wide midfielders	

	β	95%CI	β	95%CI	β	95%CI	β	95%CI	β	95%CI
TD-D										
Intercept	4162	(3844;4480)	5191	(4817;5565)	4658	(4264;5052)	4534	(3914;5154)	4746	(4268;5224)
League (N-BFLT)	**-1132**	**(-1463;-801)**	**-1636**	**(-2001;-1270)**	**-1354**	**(-1752;-955)**	**-1116**	**(-1725;-508)**	**-1412**	**(-1936;-887)**
Match outcome (not win)	-170	(-458;119)	**-344**	**(-669;-19)**	-223	(-579;133)	-261	(-929;407)	-40	(-690;609)
Location (home)	40	(-211;291)	-1	(-286;285)	50	(-253;352)	463	(-112;1037)	-51	(-489;387)

LIR-D										
Intercept	3318	(3061;3576)	3690	(3385;3994)	3310	(3033;3588)	3283	(2729;3837)	3328	(2962;3695)
League (N-BFLT)	**-987**	**(-1252;-722)**	**-1251**	**(-1550;-953)**	**-1041**	**(-1300;-782)**	**-1030**	**(-1574;-487)**	**-1218**	**(-1607;-829)**
Match outcome (not win)	-155	(-391;80)	-262	(-526;2)	-206	(-464;51)	-268	(-864;329)	-38	(-456;380)
Location (home)	129	(-76;334)	51	(-181;284)	72	(-154;298)	323	(-191;836)	39	(-284;362)

MIR-D										
Intercept	657	(571;743)	1086	(949;1223)	876	(760;991)	862	(660;1065)	843	(705;981)
League (N-BFLT)	-66	(-167;34)	**-269**	**(-411;-127)**	**-238**	**(-367;-108)**	-157	(-356;42)	-130	(-295;35)
Match outcome (not win)	-52	(-120;16)	-37	(-150;75)	-38	(-133;58)	-20	(-238;199)	-25	(-245;195)
Location (home)	-48	(-105;9)	-4	(-100;92)	11	(-66;88)	44	(-143;232)	-9	(-161;143)

HIR-D										
Intercept	197	(145;250)	394	(286;503)	468	(382;554)	394	(191;598)	572	(412;732)
League (N-BFLT)	-64	(-129;1)	-105	(-232;22)	**-100**	**(-191;-10)**	66	(-134;266)	-52	(-222;119)
Match outcome (not win)	4	(-32;41)	-25	(-97;47)	13	(-63;89)	23	(-197;242)	-12	(-194;171)
Location (home)	-29	(-58;1)	-37	(-93;20)	-8	(-72;55)	88	(-101;276)	-48	(-189;93)

β – estimate, CI – confidence interval; TD-D – total distance in defensive phase of game, LIR-D – low intensity running in defensive phase of game, MIR-D – moderate intensity running in defensive phase of game, HIR-D – high intensity running in defensive phase of game. Bold text denotes statistical significance of p < 0.05.

**TABLE 5:** Multivariate differences between N-BFLTs and BFLTs in MRP defined by discriminant canonical analysis.

	CD	CM	FB	WM	All
	Root	Root	Root	Root	Root
LIR	0.06	-0.01	-0.03	-0.15	0.05
MIR	-0.03	0.02	-0.09	-0.17	0.02
HIR	-0.20	-0.19	-0.13	0.01	-0.10
LIR-O	0.63	0.56	0.60	-0.46	0.64
RUN-O	0.19	0.33	0.39	-0.35	0.25
HIR-O	-0.14	-0.03	0.02	-0.15	0.02
LIR-D	-0.78	-0.79	-0.89	0.80	-0.87
RUN-D	-0.22	-0.38	-0.46	0.21	-0.30
HIR-D	-0.21	-0.20	-0.23	0.08	-0.14

CanR	0.81	0.85	0.79	0.89	0.80
Wilks Lambda	0.35	0.27	0.36	0.20	0.35
p	0.001	0.001	0.001	0.001	0.001

C: N-BFLT	1.03	1.17	0.96	-1.41	1.03
C: BFLT	-1.77	-2.22	-1.76	2.55	-1.79

Note: CanR – canonical correlation; Root – structure of the discriminant function/root; C – centroid; N-BFLTs – non-big five league teams, BFLTs – big five league teams; TD – total distance, LIR – low intensity running, MIR – moderate intensity running, HIR – high intensity running; TD-O – total distance in offensive phase of game, LIR-O – low intensity running in offensive phase of game, MIR-O – moderate intensity running in offensive phase of game, HIR-O – high intensity running in offensive phase of game; TD-D – total distance in defensive phase of game, LIR-D – low intensity running in defensive phase of game, MIR-D – moderate intensity running in defensive phase of game, HIR-D – high intensity running in defensive phase of game.

No differences for TD, LIR, MIR, and HIR in won and lost matches were found (all trivial ES). Lower TD-O (moderate ES), LIR-O (moderate ES) and HIR-O (small ES) were evidenced in won matches (3684 m, 2417 m, and 415, respectively) than in lost matches (4184 m, 2806 m, and 456 m, respectively). In addition, greater TD-D (moderate ES), LIR-D (moderate ES), and MIR-D (small ES) were evidenced in won matches (4009 m, 2949 m, and 743 m, respectively) than in lost matches (3433 m, 2454 m, and 671 m, respectively) (Table 6).

**TABLE 6:** Comparison of MRPs between won and lost matches

	Lost	Won	ANOVA		Effect size
	Mean±SD	Mean±SD	f	p	Cohen’s d (95%CI)
TD (m)	10,873 ± 926	10,935 ± 957	0.22	0.64	-0.06 (-0.34, 0.21)
LIR (m)	8089 ± 474	8165 ± 526	1.24	0.27	-0.14 (-0.43, 0.12)
MIR (m)	1905 ± 503	1920 ± 464	0.04	0.83	-0.03 (-0.3, 0.24)
HIR (m)	881 ± 291	853 ± 269	0.5	0.48	0.1 (-0.17, 0.37)
TD-O (m)	4184 ± 1075	3684 ± 931	12.15	0.01	0.54 (0.21, 0.76)
LIR-O (m)	2806 ± 830	2417 ± 732	12.24	0.01	0.53 (0.21, 0.76)
MIR-O (m)	922 ± 355	852 ± 278	2.28	0.13	0.25 (-0.06, 0.48)
HIR-O (m)	456 ± 145	415 ± 112	4.73	0.03	0.37 (0.03, 0.57)
TD-D (m)	3433 ± 847	4009 ± 1057	20.55	0.01	-0.54 (-0.9, -0.35)
LIR-D (m)	2454 ± 643	2949 ± 824	25.8	0.01	-0.6 (-0.98, -0.42)
MIR-D (m)	671 ± 240	743 ± 258	4.49	0.04	-0.28 (-0.57, -0.02)
HIR-D (m)	308 ± 205	317 ± 205	0.09	0.77	-0.04 (-0.32, 0.23)

Note: TD – total distance, LIR – low intensity running, MIR – moderate intensity running, HIR – high intensity running; TD-O – total distance in offensive phase of game, LIR-O – low intensity running in offensive phase of game, MIR-O – moderate intensity running in offensive phase of game, HIR-O – high intensity running in offensive phase of game; TD-D – total distance in defensive phase of game, LIR-D – low intensity running in defensive phase of game, MIR-D – moderate intensity running in defensive phase of game, HIR-D – high intensity running in defensive phase of game

Analysis of differences in ball possession between N-BFLTs and BFLTs show that N-BFLTs (mean ± SD: 42.71 ± 7.83%; range: 30–55%) had significantly lower percentage of ball possession (z = –12.12, p < 0.01) than BFLTs (mean ± SD 62.21 ± 5.51%; range: 47–70%).

## DISCUSSION

This study was the first to examine differences in MRP between the most elite soccer players (i.e., from BFLTs) and their counterparts from lower-standard teams (i.e., from N-BFLTs) who competed in the UCL while controlling for the influence of contextual factors. The main findings show that players from BFLTs, in general, achieved a similar overall MRP when compared to players from N-BFLTs, with only CDs from BFLTs achieving greater HIR than CDs from N-BFLTs in HIR. In addition, players from BFLTs on all playing positions achieved greater TD-D and LIR-D, and lower TD-O and LIR-O than players from N-BFLTs.

In pursuit of reaching elite match-play, authors have repeatedly tried to identify MRP that characterize elite players and distinguish them from their counterparts from lower-standard teams [28–31]. For example, it was reported that lower distances were covered in total- and high-intensity running (≥ 19.8 km·h-1) in higher-rated teams compared with lower-standard divisions [28, 29]. On the other hand, older studies have shown that players at a higher standard of play perform more high-intensity running than their peers at lower standards [30, 31]. The possible explanation for such contrasting findings in the literature might be the use of different methods to determine competitive levels. Moreover, none of these studies controlled for the influence of contextual factors, which have been repeatedly demonstrated to affect MRP [15]. In the current study, when the influence of match location and match outcome (i.e., as most influential contextual factors in UCL matches [11]) was controlled for, the results showed similar MRP results among the most elite UCL soccer players and their counterparts from lower-standard teams. Specifically, we found similar TD, LIR, MIR and HIR between FBs, CMs, WMs and FWs from BFLTs and N-BFLTs.

Such results indicate that MRP of the most elite and lower-standard soccer players on all playing positions do not differ, with exception for CDs. Namely, CDs from BFLTs achieved greater HIR than CDs from N-BFLTs (moderate effect size). As in general higher standard players have superior technical–tactical qualities [32, 33], it is most likely that CDs from BFLTs, although primarily defensive players, were more involved in offensive actions then their peers from N-BFLTs. Given that this requires their deeper positioning in the opponent’s half of the pitch [34], CDs from BFLTs consequently leaving larger spaces behind their backs. Considering that after losing ball in the attack phase, a rapid offensive transition by the opposing team regularly followed [35], CDs should utilize higher running speeds to outperform opponent players and successfully defend this space [34]. This may explain the increased HIR of the most elite CDs.

Taken altogether, it is evident that, overall MRPs were similar among the most elite and lower-standard soccer players. Such findings are not surprising since previous research demonstrated that MRP in the context of ball possession is a more important determinant of elite (i.e., successful) match-play than pure MRP [22]. Supportively, comparison of MRPs between won and lost matches from current study showed that successful match-play (i.e., won matches) was characterized by similar overall MRPs, decreased MRPs in offensive phase of game, and increased MRPs in defensive phase of game. Indeed, in-depth analyses of MRP conducted specifically for the offensive phase of the game (i.e., when the team has ball possession) show that players from BFLTs on all playing positions covered less TD-O and LIR-O than players from N-BFLTs (all large effect sizes), indicating that MRP of the most elite soccer players in the offensive phase of game greatly differ compared with lower-standard players. Considering that results from our study confirm previous findings that higher standard players prefer to maintain ball possession [36], while ball possession strategies consequently enables greater execution of technical-tactical performance [37], such decreased activeness in the offensive phase game among the most elite soccer players game is almost certainly consequence of their greater orientation toward the execution of technical–tactical performance, which is typically related to covering shorter distances [38].

Further analysis of MRP in offensive phase of game revealed another important finding. Specifically, FWs from BFLTs achieved less HIR-O than FWs from N-BFLTs (large effect size). This suggest that the most elite FWs in offensive phase of game did not utilize large amount of HIR. It is possible that most elite FWs in attacking phase of game actually played as “false FWs” (usually known as “false nines”) [39]. Typically, when a team has ball possession, such players drop deep into the midfield to search for the ball, which, consequently, takes opposing CDs out of their defensive line, emptying the area in which CMs or WMs should run. In such cases, FWs do not behave like classic attackers (i.e., utilizing high-intensity running in attacking actions to create goal opportunities for themselves), which, consequently, results in their lower HIR-O.

One of the most robust findings in the current study comes from an analysis of MRP in the defensive phase of the game (i.e., when opponent have ball in possession). In particular, our results show that players from BFLTs on all playing positions achieved greater TD-D and LIR-D than players from N-BFLTs (all large effect sizes), indicating that running characteristics of the most elite soccer players in the defensive phase of game greatly differ compared with lower-standard players. Taking into account previously introduced assumption that higher standard players prefer to utilize ball possession strategies [36], such increased activeness of most elite soccer players in defensive phase of game is probably consequence of their collective attempts to regain ball possession from the opponent [37]. As ball possession strategies consequently enables greater execution of technical-tactical performance [37], which are considered as essential for match success in professional soccer [17], our findings suggest that greater running efforts in the defensive phase of the game are important determinants of the most elite match-play. This can be directly supported with results of canonical discriminant analysis which show that LIR-D most greatly contributed to the differentiation between most elite soccer players (i.e., from BFLTs) and their counterparts from lower-standard teams (N-BFLTs).

It is additionally noteworthy that FBs from BFLTs achieved more MIR-O (large effect size) and HIR-O (moderate effect size) than FBs from N-BFLTs. This suggest that the most elite FBs in defensive phase of covered greater distances at moderate and high intensities, what is most likely consequence of their behaviour in offensive phase of game. Specifically, as higher standard players have superior technical–tactical qualities [32, 33], FBs from BFLTs were probably offensively more engaged than FBs from N-BFLTs. To participate more easily in offensive actions, their positioning is much deeper into the opponent’s half. On the other hand, as they are primarily defensive players, FBs travel deeply into their own half of the pitch to participate in defensive actions. Such greater involvement in offensive actions in combination of playing on a large field almost certainly results in greater physical demands (i.e., greater TD-D, LIR-D, MIR-D and HIR-D) observed in defensive phase of game [34].

### Limitations

Some limitations should be considered when interpreting these findings. A relatively small number of matches were analysed, with a limited sample of players within certain playing positions. However, this is a very common obstacle in studies involving players who compete in high-level soccer [11]. For methodological reasons, we included only players who played a whole match, which reduced the number of observations and may have affected MRP. As players’ technical-tactical performances were not available to confirm some considerations drawn from the current study, future research should analyse technical-tactical performances of the players together with MRP. Finally, for a detailed understanding of the MRPs of the most elite soccer players, future research should consider other contextual factors, such as team formations, match periods, current score, match importance and number of players on the field.

### Practical applications

Sports scientists and performance analysts use data on match running performance to mainly aid coaches and practitioners in decision-making processes for structuring the elements of training and subsequent match preparation [40]. This research suggests two main practical applications. Firstly, reaching the most elite soccer match-play require increased efforts when opponent have the ball in possession (i.e., defensive phase of game) for players on all playing positions. Therefore, physical conditioning programmes need to be adapted accordingly, with special emphasize on training drills based on re-possession of the ball. Secondly, as that overall physical performance in highest-level soccer is not related to the playing standard, while highest-level soccer is physically highly demanding [3], optimal physical preparation should be ensured for all players competing in highest-level soccer irrespective playing in higher- or lower-standard teams.

## CONCLUSIONS

The findings from this study show that the most elite soccer players on all playing positions experienced similar overall MRP as their peers from lower-standard teams. However, analysing separately offensive and defensive phase of game, noteworthy differences between the most elite soccer players and peers from lower-standard teams could be observed. Specifically, the most elite soccer players on all playing position run less in offensive phase of game, what is almost certainty consequence of their superior ball possession. On the other hand, the most elite soccer players on all playing position run more in defensive phase of game, possibly due to their greater efforts to regain ball possession. This study demonstrated that the most elite match-play in soccer is characterized by increased efforts in defensive phase of game, and decreased efforts in offensive phase of game.
